# Highly sensitive detection of ALK resistance mutations in plasma using droplet digital PCR

**DOI:** 10.1186/s12885-018-5031-0

**Published:** 2018-11-19

**Authors:** Ryohei Yoshida, Takaaki Sasaki, Yasuhiro Umekage, Sachie Tanno, Yusuke Ono, Munehiko Ogata, Shinichi Chiba, Yusuke Mizukami, Yoshinobu Ohsaki

**Affiliations:** 1 Respiratory Center, Asahikawa Medical University, 2-1-1-1 Midorigaoka-Higashi, Asahikawa, Hokkaido 078-8510 Japan; 2 Institute of Biomedical Research, Sapporo Higashi Tokushukai Hospital, Hokkaido, Japan; 3 Center for Advanced Research and Education, Asahikawa Medical University, Hokkaido, Japan; 4 Division of Gastroenterology and Hematology/Oncology, Department of Medicine, Asahikawa Medical University, Hokkaido, Japan

**Keywords:** Resistance, ALK inhibitors, Liquid biopsy, Cell-free DNA (cfDNA), Droplet digital PCR (ddPCR)

## Abstract

**Background:**

On-target resistance mechanisms found in one-third of patients receiving anaplastic lymphoma kinase (ALK) tyrosine kinase inhibitors (TKIs) are secondary ALK mutations in *ALK*-rearranged non-small cell lung cancer (NSCLC). There are large variations in the resistant mutations, unlike the epithelial growth factor receptor (EGFR) T790 M seen with the use of EGFR-TKIs. Liquid biopsy approaches using cell-free DNA (cfDNA) are used for screening and monitoring of mutations in NSCLC. However, feasible protocol for the simultaneous detection of multiple secondary ALK mutations using droplet digital PCR (ddPCR) has not been developed. An efficient strategy using cfDNA in cancer diagnostics, the development of more accurate and cost-effective tools to identify informative multiple secondary ALK mutations is clinically required.

**Methods:**

To establish a feasible assay to monitor ALK-TKI resistance mutations, we first evaluated the feasibility of ddPCR-based screening for cfDNA mutation detection of 10 distinct secondary *ALK* mutations. Positive samples were then re-analyzed using mutation-specific probes to track the growth of mutation clones with a high sensitivity.

**Results:**

Blood samples from seven *ALK*-positive patients were analyzed using the ddPCR protocol. Secondary G1202R *ALK* mutations were identified in 2 of 7 patients by the screening assay. Using the mutation-specific probes, monitoring the resistant clone during the clinical course of the disease was well demonstrated in each of the patients.

**Conclusion:**

The protocol for ddPCR-based liquid biopsy has a feasibility for the screening of secondary ALK-TKI resistance mutations and offers a tool for a cost-effective monitoring of progression in NSCLC.

**Electronic supplementary material:**

The online version of this article (10.1186/s12885-018-5031-0) contains supplementary material, which is available to authorized users.

## Background

In 2007, anaplastic lymphoma kinase (*ALK*) was discovered as a potential target in non-small cell lung cancer (NSCLC) [1]. In a small subset of NSCLC tumors, a chromosomal inversion event leads to fusion of a portion of the *ALK* gene with the echinoderm microtubule–associated protein-like 4 (EML4) gene. The resulting EML4-ALK fusion protein is constitutively active and transforming, leading to a state of oncogene addiction. *ALK* rearrangements included EML4-ALK fusions have been identified in 3–7% of patients with NSCLC (herein referred to as “*ALK*-positive” lung cancer) [2].

In patients with *ALK*-positive NSCLC, the ALK inhibitor crizotinib resulted in significant higher rates of response with an objective response rate (ORR) of 74% and a median progression-free survival (PFS) of 10.9 months than chemotherapy [3]. Multiple mechanisms of crizotinib resistance have been described in *ALK*-positive NSCLC, with secondary mutations in the ALK kinase domain having been detected in approximately 20% of patients, most commonly L1196 M (the “gate-keeper” mutation) and G1269A [4–6].

Although next-generation ALK inhibitors such as alectinib, ceritinib, and brigatinib are now available for preclinical and clinical testing, little is known about the potential mechanisms of resistance to these agents. In one small study, some patients who relapsed on ceritinib were found to have acquired new tumor mutations in the ALK kinase domain, either at position G1202 or at position F1174 [7]. The fact that the more commonly occurring crizotinib-resistant *ALK* mutations (i.e., L1196 M and G1269A) were not observed in ceritinib-resistant samples, suggesting that different patterns of resistance will emerge depending on the selective pressure of the individual ALK inhibitors for deriving resistant tumor subclones.

Preclinical studies have demonstrated that alectinib is active against the crizotinib-resistant *ALK* mutations L1196 M, C1156Y, and F1174 L, implying that alectinib may be effective in patients who have developed a resistant to crizotinib through other mechanisms [8, 9]. Gainor et al. reported the spectrum and frequency of the numerous known secondary mutations of ALK inhibitors in 103 patients with *ALK*-positive NSCLC [6], showing that a high frequency of G1202R mutation was a common feature of each ALK inhibitor. Recently, lorlatinib as a 3rd-generation ALK inhibitor has been developed and is expected to have a therapeutic effect on secondary mutations, including G1202R [10]. Although treatment strategies using multiple ALK inhibitors against secondary mutations is rapidly developing, the methods for detecting the actual causal genes in the clinic have not been established.

Tissue biopsy is a gold-standard for the molecular diagnosis of lung cancer; however, serial sampling is not feasible as a routine assessment for the recurrent tumor. Genomic (g) DNA shed from tumors into the general circulation has been intensively studied for monitoring tumor genetics and offers opportunities to trace the genomic evolution of cancer systematically [11, 12] . Sometimes referred to as “liquid biopsy”, genotyping of cell-free DNA (cfDNA) isolated from plasma and other body fluid represents an attractive non-invasive tool to monitor cancer progression and response to treatments (e.g. resistance). Given advances in sequencing technology, recent studies have demonstrated that the mutations detected by the cfDNA genotyping are highly concordant to those obtained by tumor biopsy [13, 14]. In addition, such an approach may overcome the problems associated with intratumor or intra-lesion genetic heterogeneity since the liquid biopsy using next-generation sequencing (NGS) will be able to cover the whole genetic landscape of tumor in all its complexity in near future [15].

An efficient strategy using cfDNA in cancer diagnostics, the development of more accurate and cost-effective tools to identify informative mutations is clinically required. The analysis of cfDNA using NGS is still expensive, time-consuming, and its value in clinical practice is limited. Among the new technologies for quantifying cfDNA, droplet digital PCR (ddPCR) provides the highest sensitivity for detecting and tracking actionable mutations at frequencies as low as 0.05–0.01% [16–18]. In the current study, we sought to establish a liquid biopsy protocol for ddPCR-based detection of multiple TKI-induced secondary mutations in *ALK*-positive NSCLC patients. We also describe clinical course of patients with G1202R-positive NSCLC in details.

## Methods

### Patients

Seven patients with *ALK*-positive NSCLC, previously treated or undergoing treatment, were examined between January 2015 and December 2016 at the Asahikawa Medical University Hospital, Asahikawa, Japan. All patients were diagnosed with *ALK*-positive NSCLC by fluorescence in situ hybridization (FISH) or immunohistochemistry (IHC). Patients were treated with daily crizotinib or alectinib. The Standard Response Evaluation Criteria in Solid Tumors (RECIST 1.0) was used to evaluate treatment responses. We obtained written informed consents from all study participants, and the study was approved by the Institute of Biomedical Research and Innovation Research Ethics Committee.

### Sample collection and DNA extraction

**Fig. 1:**
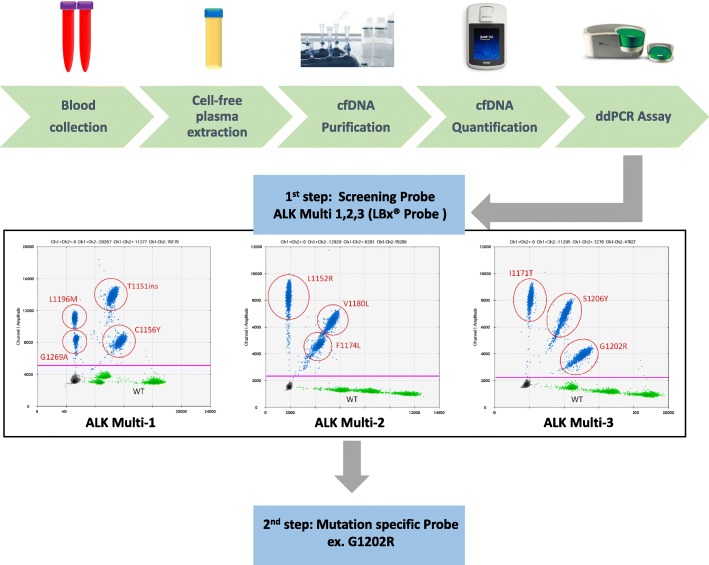
Schematic outlining the protocol used to detect recurrent *ALK* mutations from plasma-derived cfDNA. Firstly, blood collection from patients with *ALK*-positive NSCLC was performed, cell-free plasma was extracted. The cfDNA was isolated in the purification process and quantified using Qubit. We used a ddPCR genotyping assay in conjunction with the QX200 Droplet Digital PCR System. The cfDNA was analyzed in two steps. In the first step, cfDNA was analyzed using screening probes. In the second step, cfDNA mutations detected in the screening phase were re-analyzed with mutation-specific probes

Blood collection was performed every 3 months during ALK inhibitor treatment as long as blood samples were scored as being positive in the first screening step. Blood samples (limited to < 16 mL) were collected in 8 mL tubes containing EDTA-2 K (SPM-L1008EMS; Sekisui Medical, Co., Ltd.), with plasma isolated within 2 h. Tubes were centrifuged at 1,100 g for 10 min at room temperature. After the first centrifugation, tubes were centrifuged at 18,000 g for 10 min at 4 °C (Fig. 1). The supernatant, containing cell-free plasma, was collected in 2 mL serum tubes and stored at − 80 °C until purification. For the purification process, cfDNA was isolated from cell-free plasma in a volume of 2–4 mL with the QIAamp Circulating Nucleic Acid kit (Qiagen, Hilden, Germany) according to the manufacture’s protocol. Finally, cfDNA was extracted with elution buffer in volume of 100 μL. After purification, cfDNA aliquots were quantified with the Qubit dsDNA HS Assay Kit (Thermo Fisher Scientific, Waltham, MA, USA) and a Qubit 3.0 Fluorometer (Thermo Fisher Scientific).

### Detection of ALK resistance mutations in cfDNA

To detect ALK inhibitor resistance mutations in cfDNA, we used a ddPCR genotyping assay in conjunction with the QX200 Droplet Digital PCR System (Bio-Rad, Hercules, USA). The cfDNA was analyzed in two steps. In the first (screening) step, cfDNA was analyzed with the multiplex probes LBx® Probes ALK Multi-1, − 2, and − 3 (Riken genesis, Tokyo, Japan). The probe cocktails cover the ALK-TKI mutations T1151ins, C1156Y, L1196 M, G1269A (ALK Multi-1), L1152R, F1174 L, and V1180 L (ALK Multi-2), and I1171T, G1202R, and S1206Y (ALK Multi-3). The combination of these three probe sets thus allows for the detection of 10 recurrent *ALK* mutations simultaneously. In the second step, cfDNA mutations detected in the screening phase were re-analyzed with mutation-specific probes (Integrated DNA Technologies), designed to increase probe binding specificity and thus track the progression of low mutation cfDNA fractions during the clinical course. All probes in the second step were diluted to be 100 μM with indicated volume of TE Buffer.

### Droplet digital PCR system

Reaction mixtures were prepared (Additional file 1: Table S1), and partitioned into ~ 22,000 droplets per sample by mixing with 70 μL of Droplet Generation Oil (Bio-Rad) in a QX200 droplet generator (Bio-Rad). Droplets were then subjected to thermal cycling with a Veriti Thermal Cycler (Thermo Fisher Scientific) (Additional file 1: Table S2), before transferring to a QX200 droplet reader (Bio-Rad) for fluorescence measurement of 6-fluorescein amidite (FAM) and hexachloro-fluorescein (HEX) probes. Droplets were scored as positive or negative based on their fluorescence intensity, which was determined by gating thresholds, defined with positive and negative controls. Finally, absolute copy number input in the reaction and the ratio of mutated fragments was calculated by QuantaSoft ver 1.7 (Bio-Rad), based on a Poisson distribution. Samples were scored as positive for the mutation when at least two mutation droplets were detected using screening probes, or at least three mutation droplets were detected by mutation-specific ddPCR.

### Mutation-specific detection of ALK G1202R and I1171T

Probes (Integrated DNA Technologies) used in the mutation-specific assay contained locked nucleic acid (LNA) bases, which increase probe binding specificity and enable the detection of low mutation cfDNA fractions [19]. *ALK* G1202R and I1171T probe and primer sequences were designed (Additional file 1: Table S3A).

### Sensitivity and specificity of the mutation-specific ddPCR assay

To confirm the sensitivity and specificity of the specific mutations (G1202R and I1171T), we performed preliminary experiments using gBlocks® gene fragments (Integrated DNA Technologies) for wild-type and mutant *ALK* (Additional file 1: Table S3B). The *ALK* G1202R fragment was mixed with *ALK* wild-type DNA in ratios of 1:10 (10%), 1:100 (1%), 1:1,000 (0.1%), 1:10,000 (0.01%), and 1:100,000 (0.001%), and amplified with probes for wild-type *ALK* (HEX) which include both fused and non-fused of ALK and G1202R mutation (FAM) (Additional file 2: Figure S1). The limitation of detection for *ALK* G1202R was found to be 0.01% mutation DNA. % mut were calculated from generated Poisson concentrations as follows:

$$ \%\mathrm{mut}=\frac{(FAM)}{\left( FAM+ HEX\right)}\times 100. $$

### Next-generation sequencing

Genomic DNA from brain metastasis biopsy tissue (case #1) was isolated using the DNeasy Blood & Tissue Kit (Qiagen) according to the manufacturer’s protocol. Ion AmpliSeq Colon and Lung Cancer Research Panel v2 (Thermo Fisher Scientific), a previously validated panel for targeted amplicon sequencing was utilized (Thermo Fisher Scientific) [20]. Briefly, 10 nanograms of gDNA was amplified by PCR using Ion AmpliSeq Library Kit (Thermo Fisher Scientific) and the sequencing was performed on an Ion PGM System according to the manufacturer’s protocol. Sequencing reads were multiplexed, quality-filtered, and aligned to the human reference genome (GRCh37) using the Torrent Suite software (ver. 5.0.4; Thermo Fisher Scientific). Variants were identified with the Variant Caller software (ver. 5.0.4.0; Thermo Fisher Scientific). The quality of all variants called was manually confirmed by IGV software (ver. 2.3.59).

## Results

### cfDNA in ALK-positive patients

**Fig. 2:**
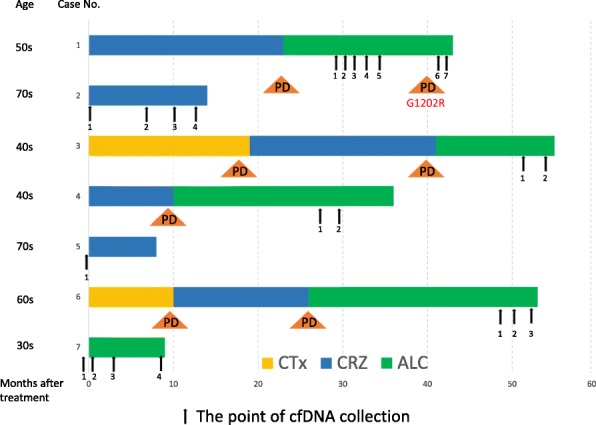
Patients characteristics; cfDNA collection from patients and treatment schedule. The timing of cfDNA collection (black allow) and each bar shows treatment period (*CTx*, chemotherapy, yellow: *CRZ* crizotinib, blue: *ALC*, alectinib, green). The number under the black arrow represents the timing of cfDNA collection in each case. cfDNA concentration and cfDNA input per well for each patient is shown in Additional file 1: Table S4. Triangle PD represents the timing of disease progression

Seven consecutive *ALK*-positive NSCLC patients were enrolled in this study. Six patients were already treated with the ALK-TKIs crizotinib or alectinib (Fig. 2 and Additional file 1: Table S4). Following cfDNA analysis in the first screening step,2 of 7 patients (28.6%) were scored as being positive. Case 1 observed ALK G1202R mutation during alectinib treatment and Case 2 also observed ALK G1202R mutation during crizotinib treatment. In the 3 patients who received alectinib treatment after crizotinib failure, tumors were still controlled (2 cases of CR and, 1 case of PR), mutation screening were negative for all 10 secondary mutations. The rest of patient was still on going with crizotinib treatment, with only obtained pre-treatment sample which was scored as negative for secondary mutations with the screening probe cocktails. In Case #1, we could successfully detect resistance mutation samples from serial blood collection during ALK-TKI treatment, also could validate the data from ddPCR and NGS analysis from tumor sample. The clinical course of the case with the acquired resistance was described below in greater detail.

### Case #1

**Fig. 3:**
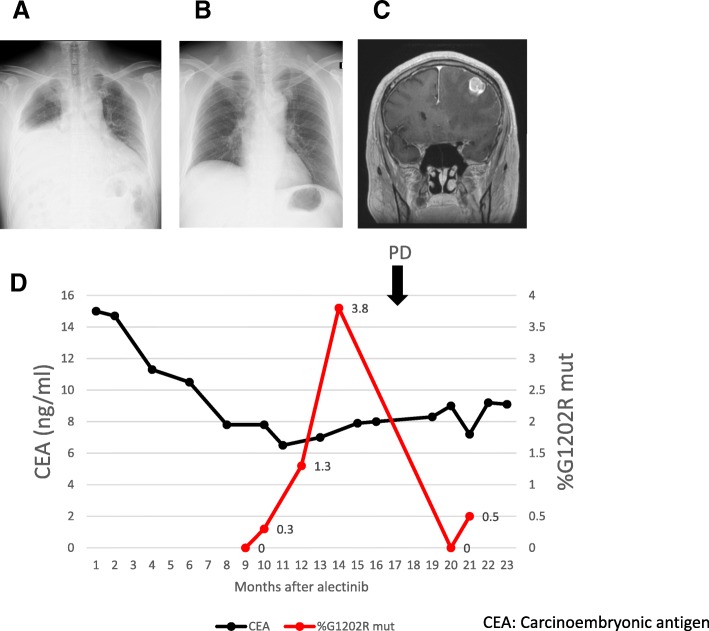
Case #1. (**a**) Chest radiography showing PD 23 months after treatment with crizotinib, with pericardial effusion and right pleural effusion. (**b**) Chest radiography 13 months after treatment with alectinib. Imaging revealed a moderate response in pericardial effusion and right pleural effusion. (**c**) MRI imaging showing PD (brain metastasis) 17 months after treatment with alectinib. (**d**) Graph shows the change of % G1202R mut in cfDNA using ddPCR and CEA levels during the clinical course after treatment with alectinib. % G1202R mut $=\frac{\left( FAM\ \right)}{\left(\  FAM+ HEX\ \right)}$ ×100. PD = Progressive disease

A Japanese in his fifties with a history of NSCLC, that had undergone right upper lobe resection and mediastinal lymph node dissection, was diagnosed with postoperative recurrence in lung harboring an *ALK*-rearrangement by FISH. After 23 months treatment with crizotinib, pericardial effusion and right pleural effusion were found (Fig. 3a & b). Following diagnosis of progressive disease on crizotinib, medication was changed to the alternative ALK-TKI alectinib. The patient initially maintained partial clinical response on alectinib therapy, but brain metastasis was developed 17 months later (Fig. 3c). gDNA extracted from the brain tumor biopsy was sequenced and G1202R *ALK* mutation was found (variant allele frequency 26.5%; Additional file 2: Figure S2A). The recurrent *ALK* G1202R mutation in the patient matched at the nucleotide levels with the gBlock used as the *ALK* G1202R positive control.

Serial blood samples taken at seven points following the alectinib therapy (6, 8, 9, 11, 13, 19 and 20 months) were analyzed by the ddPCR screening protocol as described in the Materials and Methods. The G1202R mutation was identified with the ALK Multi-3 assay, being first detectable 11 months after the alectinib therapy (Additional file 2: Figure S2B). No *ALK* mutations were detected using ALK Multi-1 or ALK Multi-2 probes.

Subsequently, these samples were re-analyzed using the mutation-specific assay. The G1202R mutation was detected 9 months after the therapy and the mutant fraction was increased over time (Fig. 3d). In contrast, CEA levels remained above the reference value (5 ng/ml) during the observation period and alectinib failure was not demonstrated. The time between the plasma cfDNA-based progressive disease and recurrence of the brain metastasis was 8 months. After resection of the brain metastasis, the frequency of G1202R mutation decreased. The patient was treated with alectinib as beyond PD therapy, but there were no progression on alectinib five months after resection of the brain metastasis (Fig. 3d & Additional file 2: Figure S3C).

## Discussion

In this study, we established a protocol for liquid biopsy-based ddPCR detection of multiple secondary mutations in *ALK*-positive NSCLC patients. In the first screening step, we can identify multiple *ALK* mutations with high sensitivity, while in the second mutation-specific step we are able to monitor disease progression and thus ALK-TKI clinical efficacy. Using this protocol, we report two cases in which the *ALK* mutation G1202R emerged as a mechanism of resistance to ALK-TKIs or G1202R mutation detected during treatment with crizotinib and it was not associated with resistance to this drug.

Recently, liquid biopsy using cfDNA for screening or monitoring of response to treatment has become more widely used, with studies reporting a concordance of 70–80% between tissue and plasma mutations identified in NSCLCs [21, 22]. Although this figure indicates that there is still room for improvement, liquid biopsy approaches using cfDNA have the potential to address some important clinical problems. One is the issue of intra-tumor heterogeneity, wherein the population of mutated, resistant and wildtype clones may change over time under the selection pressure of targeted therapy [23]. Another problem is the challenge of performing multiple and potentially difficult biopsies, such as those involving bronchoscopy, for the purpose of monitoring disease progression. In this study, we have established an effective protocol for liquid biopsy-based ddPCR detection of multiple ALK-TKI resistance mutations in *ALK*-positive NSCLC patients. In this protocol, multiplex probes ALK Multi1, − 2, and − 3 were initially used for screening, with mutation-specific probes (e.g., for G1202R) then used to monitor disease progression.

In contrast to ALK-TKI resistance, in which many secondary mutations have been reported [5, 24], half of the acquired resistance to epithelial growth factor receptor (EGFR) TKI therapy has been shown to be due acquisition of a single point mutation T790 M [24]. Consequently, many strategies have been developed to detect T790 M mutations with high sensitivity. The amplification-refractory mutation system (ARMS) is one such assay that has been developed to detect mutations using cfDNA in the blood. Wang et al. reported that, comparing ddPCR with ARMS in 75 patient plasma samples, the rates of T790 M mutation detection were 46.7% by ddPCR, and 25.3% by ARMS [25]. This result suggests that ddPCR assay may provide greater sensitivity than ARMS to detect the *EGFR* T790 M mutation in plasma. In the present study, we were similarly able to detect *ALK* mutations in plasma-derived cfDNA using a highly sensitive ddPCR approach. Since it is known that multiple secondary mutations may be involved in the development of resistance to ALK-TKIs [5, 24], it is important to screen *ALK*-positive patients for a wide-range of potential mutations, as we have described here. The mutation-specific G1202R assay that we designed contained LNA, which is a high-affinity DNA analogue that hybridizes to cDNA and this chemistry contributes to an improvement in detection specificity [26]. Importantly, we were able to use this protocol to identify two NSCLC patients who developed G1202R point mutations as a mechanism of resistance to ALK-TKIs or G1202R mutation detected during treatment with crizotinib and it was not associated with resistance to this drug. In Case No. 1, especially, the plasma levels of the *ALK* mutation corresponded with the patient’s clinical course, indicating that this is an approach that has clinical utility for the monitoring of therapeutic effect. ALK G1202R has been shown to confer resistance to crizotinib and second-generation ALK inhibitors in both preclinical and clinical studies [4–6, 27]. This is the first case who treated with crizotinib harboring G1202R mutation. EML4-ALK protein thought to generate dimerization or trimerization which causes phosphorylation of ALK protein. The ALK TKI potency difference between ALK-WT/ALK-G1202R heterodimer or ALK-WT/ALK-G1202R/ALK-G1202R hetero-trimer and ALK-G1202R homodimer or ALK-G1202R trimer may be one of the explanation [28, 29]. There is a limitation of our study using this protocol. Our results showed that only limited number of patients and were analyzed in a single institutional experience. In order to further validation, we are planning multi-institutional study.

## Conclusions

This is the first protocol described for the liquid biopsy-based ddPCR detection of multiple ALK-TKI secondary resistance mutations in *ALK*-positive NSCLC patients. A limitation of this study is that the number of patient samples was small; however, the results indicate that there may be clinical benefit in monitoring the emergence of resistance mutations to provide early evidence of disease progression in NSCLC. A long-term surveillance study in a larger cohort of patients is now warranted to confirm these important findings.

## Additional files

Additional file 1: **Table S1.** Reaction mixtures. (A) Screening assay. (B) Mutant specific assay. **Table S2.** Thermal cycling conditions. (A) Screening assay. (B) Mutant specific assay. **Table S3.** Mutation-specific detection. (A) ALK G1202R and I1171T probe and primer. (B) gblocks® gene fragments. **Table S4.** cfDNA concentration and cfDNA input volume for patients. (DOCX 20 kb)

Additional file 2:**Figure S1.** Sensitivity and specificity of mutant specific assay for the ALK G1202R mutation. (A) Ch1 amplitude using G1202R mutation probe (FAM); (B) Ch2 amplitude using Wt probe (HEX); (C) Copy number of positive droplets for the G1202R mutation (Mt, blue bar) and wildtype (Wt, green bars), with the red numbers indicating %G1202R mut $=\frac{\left( FAM\ \right)}{\left(\  FAM+ HEX\ \right)}$ ×100. gblocks, Mutant G1202R (Mt) and wildtype (Wt) DNA was mixed at ratios of 1:10 (10%), 1:100 (1%), 1:1000 (0.1%), 1:10000 (0.01%), and 1:100000 (0.001%), while mutant I1171T and wildtype DNA was mixed at a ratio of 1:100 (1%) (rightmost bar). **Figure S2.** (A) Next-generation sequencing analysis of the brain metastasis sample of Case 1. Brain metastasis biopsy tissue was analyzed using next-generation sequencing. The picture was taken with the Integrative Genomics Viewer. In this picture, targeted NGS identified an acquired C → T mutation in 26.5% of reads, encoding for an ALK G1202R mutation (COSM144250). (B) Two dimensions graph shows plot of the ddPCR count of plasma *ALK* mutation using ALK Multi3 probe in the first screening step. The patient plasma sample surrounded by the red circle was taken 11 months after alectinib treatment. The groups surrounded by black circles show each positive control (mutation I1171T, blue; G1202R, orange). Ch1 amplitude using G1202R mutation probe (FAM); Ch2 amplitude using Wt probe (HEX). (C) Ch1 amplitude using G1202R mutation probe (FAM); Ch2 amplitude using Wt probe (HEX); Copy number of positive droplets for the G1202R mutation (Mt, blue bar) and wildtype (Wt, green bars), with the red numbers indicating %G1202R mut $=\frac{\left( FAM\ \right)}{\left(\  FAM+ HEX\ \right)}$ ×100. ddPCR count of plasma *ALK* G1202R mutation and correlation with clinical course. Patient samples were taken at seven points after administration of alectinib (6, 8, 9, 11, 13, 19 and 20 month). **Figure S3.** (A) Two dimensions graph shows plot of the ddPCR count of plasma *ALK* mutation using ALK Multi3 probe in the first screening step. The patient plasma sample surrounded by the red circle was taken at 1 month after crizotinib treatment. The groups surrounded by black circles show each positive control (mutation I1171T, blue; G1202R, orange). (B) Ch1 amplitude using G1202R mutation probe (FAM); Ch2 amplitude using Wt probe (HEX); Copy number of positive droplets for the G1202R mutation (Mt, blue bar) and wildtype (Wt, green bars), with the red numbers indicating %G1202R mut $=\frac{\left( FAM\ \right)}{\left(\  FAM+ HEX\ \right)}$ ×100. Correlation of the ddPCR count of plasma *ALK* G1202R mutation with clinical course. Patient samples were taken at four points after administration of alectinib; 1, 7, 11, 13 months. (PPTX 4319 kb)

## Abbreviations

ALK: anaplastic lymphoma kinase
ARMS: amplification-refractory mutation system
CEA: Carcinoembryonic antigen
cfDNA: cell-free DNA
ddPCR: droplet digital PCR
EGFR: epithelial growth factor receptor
EML4: echinoderm microtubule–associated protein-like 4
FAM: fluorescence measurement of 6-fluorescein amidite
FISH: fluorescence in situ hybridization
HEX: hexachloro-fluorescein
IHC: immunohistochemistry
LNA: locked nucleic acid
NGS: next-generation sequencing
NSCLC: non-small cell lung cancer
ORR: objective response rate
PFS: progression-free survival
TKIs: tyrosine kinase inhibitors
