# Supplementary material for: Highly sensitive detection of ALK resistance mutations in plasma using droplet digital PCR
Source: BMC Cancer. 2018 Nov 19;18:1136. doi: 10.1186/s12885-018-5031-0 (PMC6245722; doi:10.1186/s12885-018-5031-0)
Supplement: Supplementary file 1 — Table S1. Reaction mixtures. (A) Screening assay. (B) Mutant specific assay. Table S2. Thermal cycling conditions. (A) Screening assay. (B) Mutant specific assay. Table S3. Mutation-specific detection. (A) ALK G1202R and I1171T probe and primer. (B) gblocks® gene fragments. Table S4. cfDNA concentration and cfDNA input volume for patients. (DOCX 20 kb) [file 12885_2018_5031_MOESM1_ESM.docx]

**Supplemental Materials**

Supplementary Table 1. Reaction mixtures

(A) Screening assay

| Component | Volume (µL) |
| --- | --- |
| LBx® Probe ALK Multi | 2 |
| 2Bx® ProSupermix for Probes (no dUTP) | 10 |
| DNA sample | 5 |
| Water | 5 |
| Final volume | 22 |

(B) Mutant specific assay

| Component | Volume (µL) |
| --- | --- |
| ALK G1202R Probe; Wildtype probe (HEX) 100μM | 0.5 |
| ALK G1202R Probe; Alternative probe (FAM) 100μM | 0.5 |
| ALK G1202R Primer forward | 1 |
| ALK G1202R Primer reverse | 1 |
| 2LK G120Supermix for Probes (no dUTP) | 10 |
| DNA sample | 5 |
| Water | 4 |
| Final volume | 22 |

Supplementary Table 2. Thermal cycling conditions

(A) Screening assay

| Temperature (°C) | Time (min) | Ramp Rate (°C/sec) | Cycle |
| --- | --- | --- | --- |
| 95 | 10 | 2 | 1 |
| 94 | 0.5 |  | 40 |
| 58 | 1 |  |  |
| 98 | 10 |  | 1 |
| 4 | ∞ |  | 1 |

(B) Mutant specific assay

| Temperature (°C) | Time (min) | Ramp Rate (°C/sec) | Cycle |
| --- | --- | --- | --- |
| 95 | 10 | 2 | 1 |
| 94 | 0.5 |  | 40 |
| 57 | 1 |  |  |
| 98 | 10 |  | 1 |
| 4 | ∞ |  | 1 |

Supplementary Table 3. Mutation-specific detection

1. ALK G1202R and I1171T probe and primer

| G1202R |  |
| --- | --- |
| Probe |  |
| wild type (HEX) | AGGTCT+C+C+CCC |
| alternative (FAM) | AGGTCT+C+T+C+CC |
| Primer |  |
| Forward | 5’-GTGAGCCCTGCAATCCCT-3’ |
| Reverse | 5’-AAAGACTGGTTCTCACTCACC-3’ |

| I1171T |  |
| --- | --- |
| Probe |  |
| wild type (HEX) | TTA+C+CT+G+A+TGAT+CAG |
| alternative (FAM) | TA+CCT+G+G+TGA+TC |
| Primer |  |
| Forward | 5’-CTGAACAGGACGAACTGGATT-3’ |
| Reverse | 5’-AAACCTCTCCAGGTTCTTTGG-3’ |

1. gblocks® gene fragments

| mutation G1202R (Mt) | CAAATTCAACCACCAGAACATTGTTCGCTGCATTGGGGTGAGCCTGCAATCCCTGCCCCGGTTCATCCTGCTGGAGCTCATGGCGGGGAGAGACCTCAAGTCCTTCCTCCGAGAGACCCGCCCTCGCCCG |
| --- | --- |
| wildtype *ALK* (Wt) | CAAATTCAACCACCAGAACATTGTTCGCTGCATTGGGGTGAGCCTGCAATCCCTGCCCCGGTTCATCCTGCTGGAGCTCATGGCGGGGGGAGACCTCAAGTCCTTCCTCCGAGAGACCCGCCCTCGCCCG |
| mutation I1171T | ACGCTGCCTGAAGTGTGCTCTGAACAGGACGAACTGGATTTCCTCATGGAAGCCCTGATCACCAG |

Supplementary Table 4. cfDNA concentration and cfDNA input volume for patients

| Case No. | cfDNA(ng/ml) (Input cfDNA(ng)) | | | | | | |
| --- | --- | --- | --- | --- | --- | --- | --- |
| cfDNA collection | 1 | 2 | 3 | 4 | 5 | 6 | 7 |
| 1 | 8.26 (1.61) | 11.4(2.28) | 11.9 (2.84) | 8.55 (1.71) | 2.65 (0.53) | 18.4 (4.05) | 10.5 (2.98) |
| 2 | 78 (13.7) | 9.23 (2.03) | 19.7 (3.93) | 23.4 (3.98) |  |  |  |
| 3 | 12.2 (1.77) | 8 (1.6) |  |  |  |  |  |
| 4 | 8.16 (1.51) | 15.9 (1.99) |  |  |  |  |  |
| 5 | 11.7 (2.33) |  |  |  |  |  |  |
| 6 | 9.51 (1.76) | 17 (3.4) | 4.95 (0.965) |  |  |  |  |
| 7 | 6.28 (1.22) | 6.28 (1.44) | 5.4 (1.08) | 6.02 (1.84) |  |  |  |
