# Supplementary material for: Highly sensitive detection of ALK resistance mutations in plasma using droplet digital PCR
Source: BMC Cancer. 2018 Nov 19;18:1136. doi: 10.1186/s12885-018-5031-0 (PMC6245722; doi:10.1186/s12885-018-5031-0)
Supplement: Supplementary file 2 — Figure S1. Sensitivity and specificity of mutant specific assay for the ALK G1202R mutation. (A) Ch1 amplitude using G1202R mutation probe (FAM); (B) Ch2 amplitude using Wt probe (HEX); (C) Copy number of positive droplets for the G1202R mutation (Mt, blue bar) and wildtype (Wt, green bars), with the red numbers indicating %G1202R mut $=\frac{\left( FAM\ \right)}{\left(\ FAM+ HEX\ \right)}$=FAMFAM+HEX ×100. gblocks, Mutant G1202R (Mt) and wildtype (Wt) DNA was mixed at ratios of 1:10 (10%), 1:100 (1%), 1:1000 (0.1%), 1:10000 (0.01%), and 1:100000 (0.001%), while mutant I1171T and wildtype DNA was mixed at a ratio of 1:100 (1%) (rightmost bar). Figure S2. (A) Next-generation sequencing analysis of the brain metastasis sample of Case 1. Brain metastasis biopsy tissue was analyzed using next-generation sequencing. The picture was taken with the Integrative Genomics Viewer. In this picture, targeted NGS identified an acquired C → T mutation in 26.5% of reads, encoding for an ALK G1202R mutation (COSM144250). (B) Two dimensions graph shows plot of the ddPCR count of plasma ALK mutation using ALK Multi3 probe in the first screening step. The patient plasma sample surrounded by the red circle was taken 11 months after alectinib treatment. The groups surrounded by black circles show each positive control (mutation I1171T, blue; G1202R, orange). Ch1 amplitude using G1202R mutation probe (FAM); Ch2 amplitude using Wt probe (HEX). (C) Ch1 amplitude using G1202R mutation probe (FAM); Ch2 amplitude using Wt probe (HEX); Copy number of positive droplets for the G1202R mutation (Mt, blue bar) and wildtype (Wt, green bars), with the red numbers indicating %G1202R mut [file 12885_2018_5031_MOESM2_ESM.pptx]

## Slide 1
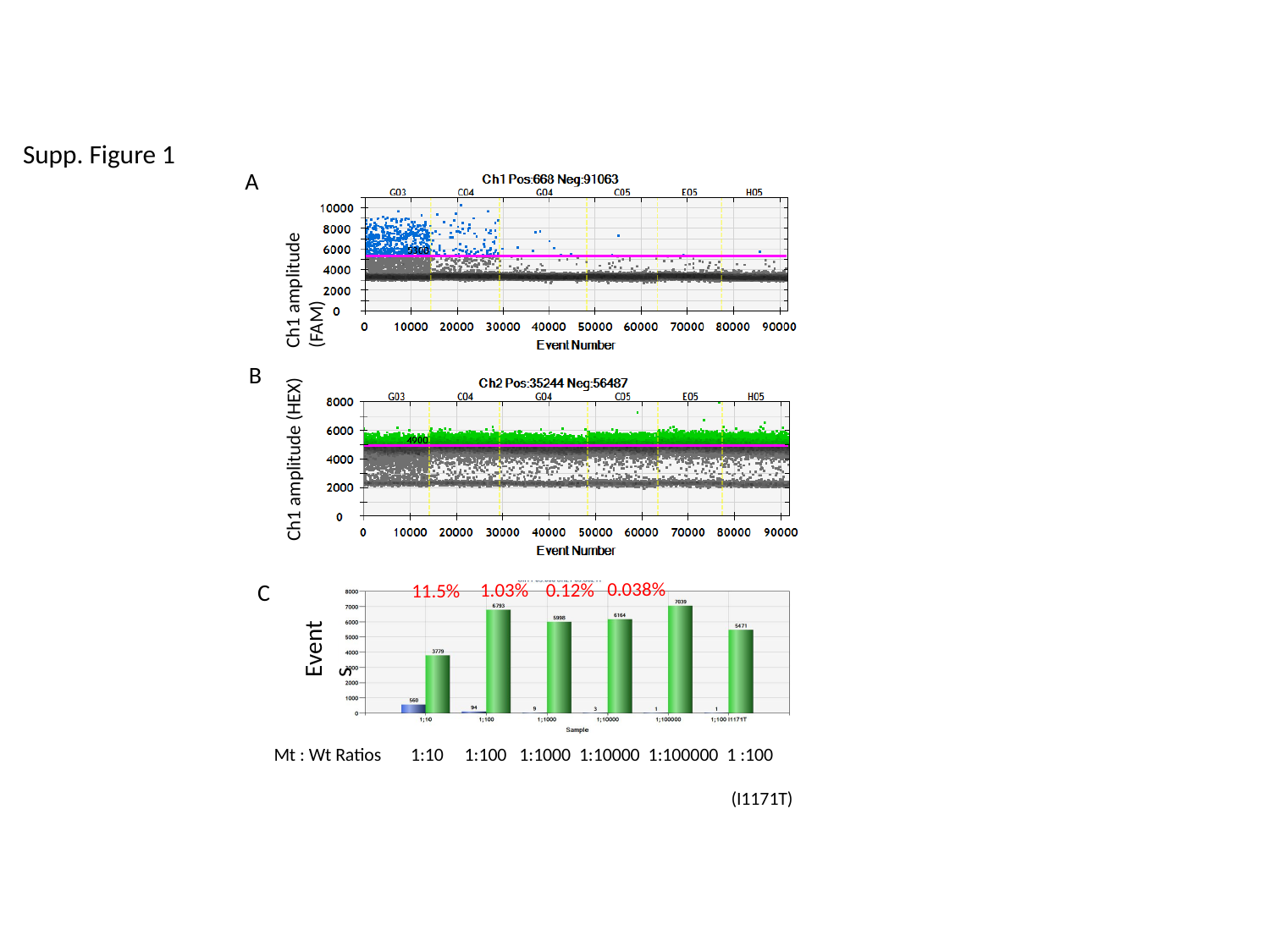

Supp. Figure 1
A
Ch1 amplitude (FAM)
B
Ch1 amplitude (HEX)
0.038%
0.12%
C
1.03%
11.5%
Events
Mt : Wt Ratios 1:10 1:100 1:1000 1:10000 1:100000 1 :100
 (I1171T)

## Slide 2
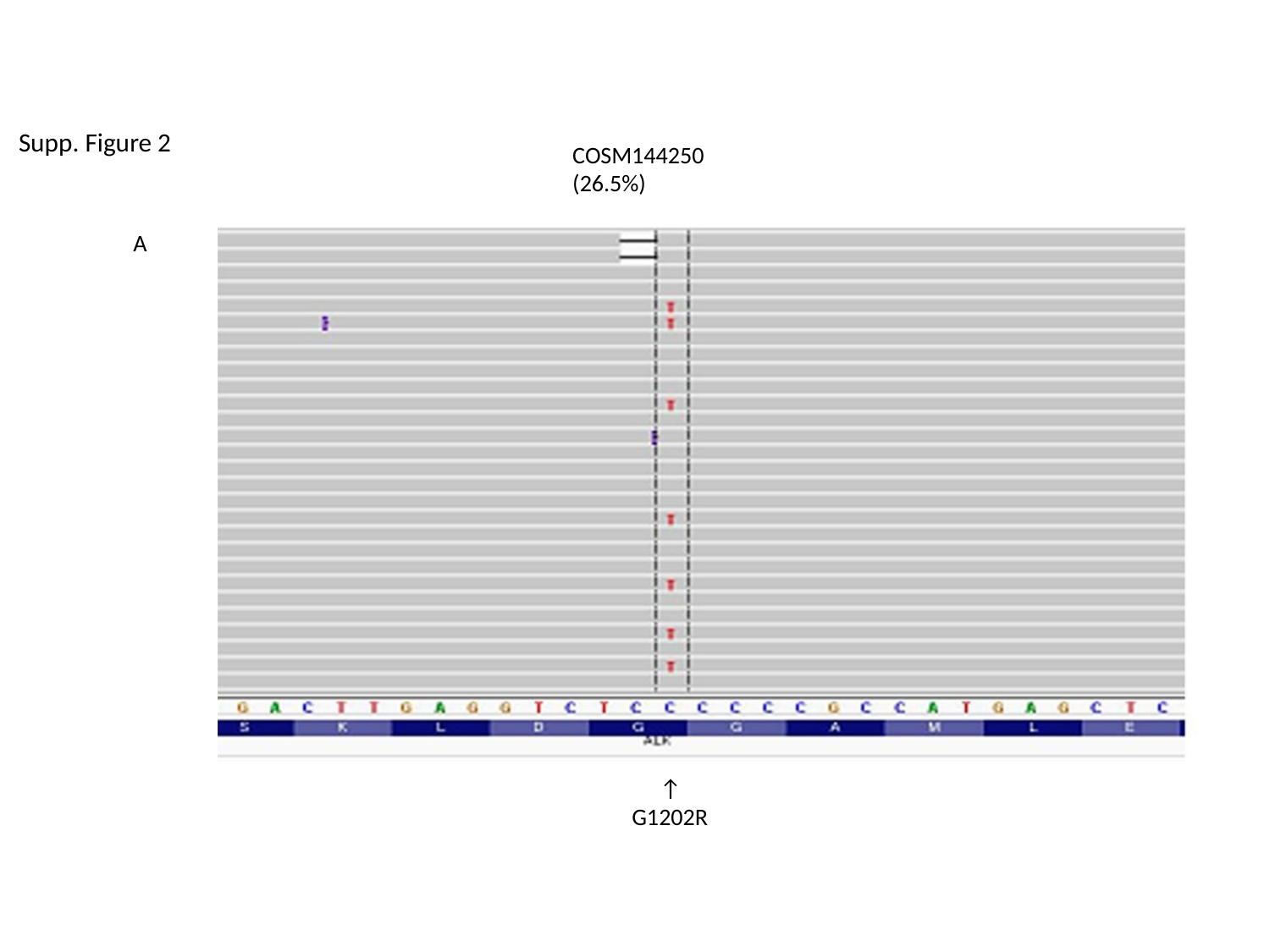

Supp. Figure 2
COSM144250 (26.5%)
A
 ↑
G1202R

## Slide 3
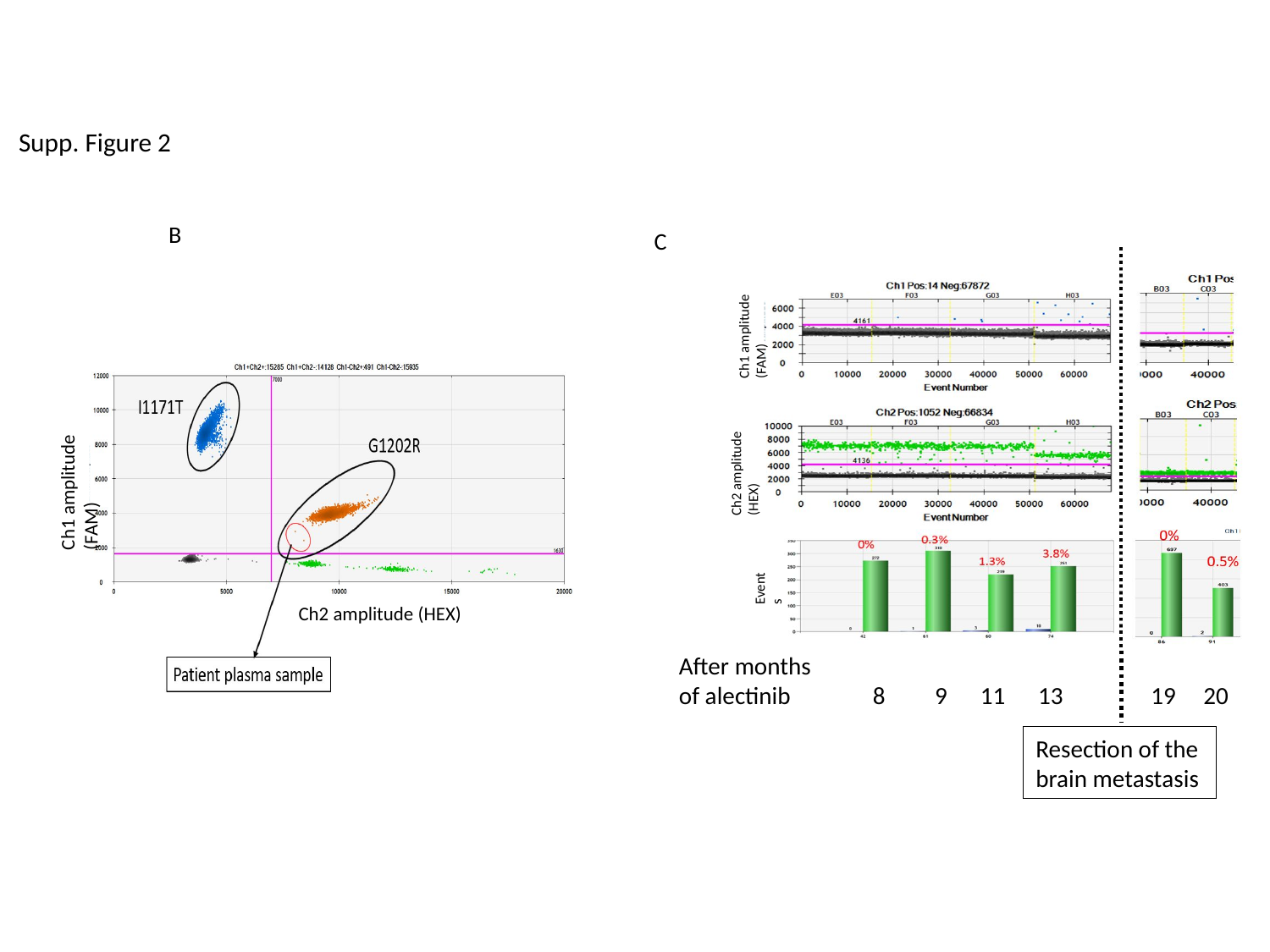

Supp. Figure 2
B
C
Ch1 amplitude (FAM)
Ch1 amplitude (FAM)
Ch2 amplitude (HEX)
Events
Ch2 amplitude (HEX)
After months
of alectinib 8 9 11 13 19 20
Resection of the brain metastasis
